# Investigation of the Interaction of Human Origin Recognition Complex Subunit 1 with G-Quadruplex DNAs of Human *c-myc* Promoter and Telomere Regions

**DOI:** 10.3390/ijms22073481

**Published:** 2021-03-27

**Authors:** Afaf Eladl, Yudai Yamaoki, Shoko Hoshina, Haruka Horinouchi, Keiko Kondo, Shou Waga, Takashi Nagata, Masato Katahira

**Affiliations:** 1 Institute of Advanced Energy, Kyoto University, Kyoto 611-0011, Japan; afaf.eladl.82m@st.kyoto-u.ac.jp (A.E.); yamaoki.yudai.7n@kyoto-u.ac.jp (Y.Y.); kondo.keiko.3u@kyoto-u.ac.jp (K.K.); nagata.takashi.6w@kyoto-u.ac.jp (T.N.); 2 Graduate School of Energy Science, Kyoto University, Kyoto 606-8501, Japan; 3 Department of Microbiology and Immunology, Faculty of Pharmacy, Zagazig University, Zagazig 44519, Egypt; 4 Department of Chemical and Biological Sciences, Japan Women’s University, Tokyo 112-8681, Japan; shoko-h@med.kitasato-u.ac.jp (S.H.); m1417081hh@ug.jwu.ac.jp (H.H.); swaga@fc.jwu.ac.jp (S.W.)

**Keywords:** origin recognition complex, G-quadruplex, DNA replication, replication origin, NMR, structure

## Abstract

Origin recognition complex (ORC) binds to replication origins in eukaryotic DNAs and plays an important role in replication. Although yeast ORC is known to sequence-specifically bind to a replication origin, how human ORC recognizes a replication origin remains unknown. Previous genome-wide studies revealed that guanine (G)-rich sequences, potentially forming G-quadruplex (G4) structures, are present in most replication origins in human cells. We previously suggested that the region comprising residues 413–511 of human ORC subunit 1, hORC1^413–511^, binds preferentially to G-rich DNAs, which form a G4 structure in the absence of hORC1^413–511^. Here, we investigated the interaction of hORC1^413-511^ with various G-rich DNAs derived from human *c-myc* promoter and telomere regions. Fluorescence anisotropy revealed that hORC1^413–511^ binds preferentially to DNAs that have G4 structures over ones having double-stranded structures. Importantly, circular dichroism (CD) and nuclear magnetic resonance (NMR) showed that those G-rich DNAs retain the G4 structures even after binding with hORC1^413–511^. NMR chemical shift perturbation analyses revealed that the external G-tetrad planes of the G4 structures are the primary binding sites for hORC1^413–511^. The present study suggests that human ORC1 may recognize replication origins through the G4 structure.

## 1. Introduction

DNA replication starts from multiple chromosomal loci called replication origins. Origin recognition complex (ORC) binds to a replication origin and recruits other replication factors [1]. ORC composed of six subunits (ORC1-6) is highly conserved in all eukaryotes and plays a crucial role in the initiation of DNA replication. It is well-known that the ORC of *Saccharomyces cerevisiae* recognizes an origin through sequence-specific binding to autonomously replicating sequences [2,3]. However, human ORC (hORC) binds to a replication origin without sequence specificity and how hORC recognizes the origin remains unclear [4,5,6,7].

Recently, the structures of yeast [8,9,10], fly [11,12], and human [13,14,15,16] ORCs in free and complex forms with either double-stranded DNAs (dsDNAs) or other replication factors were determined by X-ray crystallography, NMR spectroscopy, and cryo-electron microscopy. These structures provided critical insights into how ORC subunits, dsDNAs, and other replication factors interact with each other. Nevertheless, the mechanism of the origin recognition by hORC is still unknown.

It is well-known that guanine (G)-rich DNAs containing continuous stretches of guanine residues can fold into a four-stranded structure, the G-quadruplex (G4) structure. The G4 structure is formed through stacked guanine-tetrad (G-tetrad) planes composed of four guanine residues. So far, genome-wide analysis has revealed that more than 60% of fly, mouse, and human origins contain G-rich sequences that are potentially G4-forming [17,18,19,20]. It was revealed that the deletion of G-rich sequences causes functional impairment of replication origins and the insertion of G-rich sequences creates a new replication origin [21,22]. These results suggested that the G4 structure plays a critical role in the initiation of DNA replication.

It was reported that replication starts in the 5′ flanking DNA of the *c-myc* gene in multiple species, including human [23]. It was shown by combining chromatin immunoprecipitation assays with sequencing (ChIP-Seq) that hORC1, one of the subunits in hORC, binds to a region around the transcription start site (TSS), involving a promoter region [4]. Many promoter regions of oncogenes contain G-rich sequences that can potentially form G4 structures [24]. More recently, it was indicated that some of those G-rich sequences form G4 structures in a chromatin [25,26]. These findings suggest that G4 structures in the promoter region may be used for binding by hORC for initiation of replication. 

Previously, we found that hORC binds more strongly to DNAs having G4 structures than to those having double-stranded structures [27]. We also found that a region comprising residues 413–511 of hORC1 (hORC1^413–511^) is responsible for binding to nucleic acids, which form G4 structures in the absence of hORC. 

Here, we examined the interactions of hORC^413–511^ with DNAs, derived from *c-myc* promoter and telomere regions, having G4 structures. wtPu22 and wtPu19 (Table 1) were identified previously as nuclease hypersensitive element III_1_ (NHE III_1_) of the human *c-myc* promoter region [28]. wtPu22 and wtPu19 were revealed to dominantly fold into a parallel-type G4 structure [29]. Their mutants, mtPu22 and mtPu19 (Table 1), were revealed to fold exclusively into a parallel-type G4 structure [28,30,31]. TeloDNA (Table 1) was derived from a sequence of the human telomere region, (TTAGGG)_n_ [32]. G4 structures formed from a telomeric sequence have been well-studied and the NMR structure of teloDNA was reported to be the (3+1)-type G4 structure [32]. Firstly, we examined the binding of hORC^413–511^ to DNAs having G4 structures using fluorescence anisotropy (FA). Secondly, we examined whether the G4 structures of these DNAs are retained on binding of hORC^413–511^. Thirdly, the sites of DNAs with which hORC^413–511^ interacts were identified. Our findings suggest that the G4 structures of DNAs may be used for the recognition of replication origins by ORC.

## 2. Results

### 2.1. Binding of hORC1^413–511^ to DNAs Having a G4 Structure

**Figure 1:**
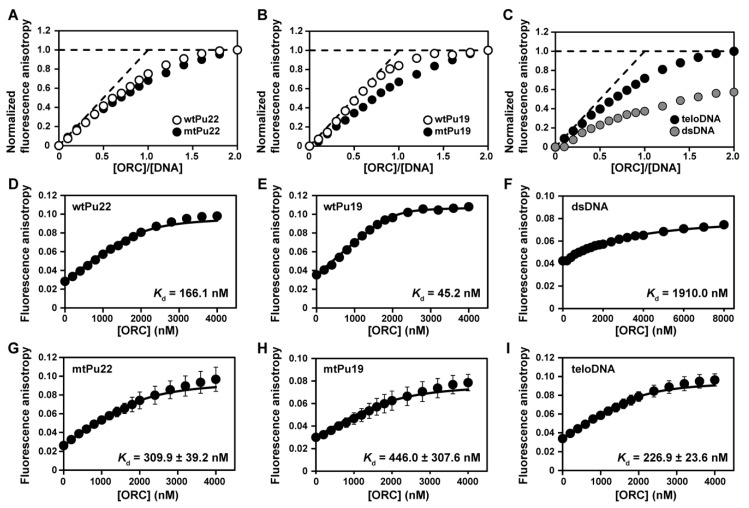
Fluorescence anisotropy (FA) titration curves of FAM-labeled G-rich DNAs derived from *c-myc* promoter or telomere regions against human origin recognition complex containing residues 413–511 (hORC1^413–511^). Changes in the normalized FA of FAM-labeled wtPu22 (open) and mtPu22 (solid) (**A**), those of FAM-labeled wtPu19 (open) and mtPu19 (solid) (**B**), and those of FAM-labeled dsDNA (grey) and teloDNA (black) (**C**) upon titration. Titration curves were fitted by using Equations (1) and (2) for FAM-labeled wtPu22 (**A**,**D**), for FAM-labeled wtPu19 (**B**,**E**), for FAM-labeled dsDNA (**C**,**F**), for FAM-labeled mtPu22 (**A**,**G**), for FAM-labeled mtPu19 (**B**,**H**), and for FAM-labeled teloDNA (**C**,**I**). The experiments in panels G, H, and I were performed in triplicate and the standard deviation is indicated for each point.

Firstly, bindings of hORC1^413–511^ to G-rich DNAs were characterized by monitoring the change in the fluorescence anisotropy (FA) of fluorescein (FAM)-labeled G-rich DNAs upon titration with hORC1^413–511^. The FA of all FAM-labeled G-rich DNAs increased with increasing molar ratio of [hORC1^413–511^]/[FAM-DNA (Figure 1A–C). wtPu22 and mtPu22, and wtPu19 and mtPu19 showed similar binding curves to each other, respectively, upon hORC1^413–511^ binding (Figure 1A,B). Additionally, teloDNA showed similar binding curves with these DNAs (Figure 1A–C). These results indicate that hORC1^413–511^ can bind to DNAs having a G4 structure, including DNAs naturally occurring in the *c-myc* promoter and telomere regions. Importantly, the binding of hORC1^413–511^ to DNAs having a G4 structure is stronger than its binding to dsDNA (Figure 1A–C). This finding is consistent with our previous report [27].

On analysis of the intersection point of an initial slope line and a plateau line (dashed lines in Figure 1A–C) of the binding curves, hORC1^413–511^ exhibited 1:1 binding stoichiometry as to all the G-rich DNAs. We deduced the dissociation constant (*K*_d_) of the complex of hORC1^413–511^ with each DNA using a 1:1 binding model (Equations (1) and (2) in Materials and Methods). The obtained *K*_d_ values were 166.1 nM for wtPu22, 45.2 nM for wtPu19, 1910.0 nM for dsDNA, 309.9 ± 39.2 nM for mtPu22, 446.0 ± 307.6 nM for mtPu19, and 226.9 ± 23.6 nM for teloDNA (Figure 1D–I). Importantly, hORC1^413–511^ bound to all the DNAs having G4 structures with higher binding affinities than the dsDNA. Since wtPu22 and wtPu19 take multiple G4 structures, and mtPu22 and mtPu19 take a single parallel-type G4 structure, we used the latter DNAs for further experiments.

**Figure 2:**
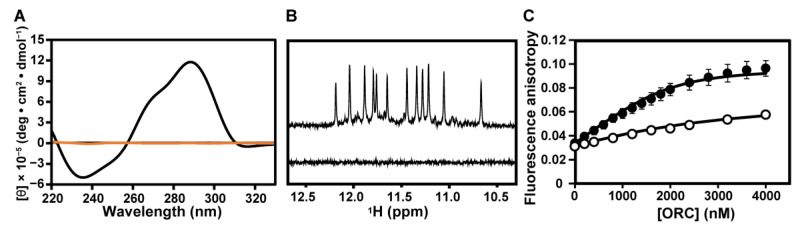
Effects of KCl and LiCl on the structure formation of teloDNA and binding of teloDNA to hORC1^413–511^. (**A**) CD spectra of teloDNA under 100 mM KCl (black) and 100 mM LiCl (orange) conditions. (**B**) ^1^H NMR spectra of teloDNA under 100 mM KCl (top) and 100 mM LiCl (bottom) conditions. (**C**) FA titration curve of FAM-labeled teloDNA against hORC1^413–511^ under 100 mM KCl (filled) and 100 mM LiCl (open) conditions.

Next, we examined the necessity of the G4 structure for the binding. It is well-known that monovalent cations, such as K^+^, stabilize G4 structure but not Li^+^ [33]. Under 100 mM KCl conditions, teloDNA produced positive peaks at 267 and 290 nm in the CD spectrum (Figure 2A, black). This indicated the formation of a (3+1)-type G4 structure [34]. Under 100 mM LiCl conditions, these peaks were absent, which indicated no formation of the G4 structure (Figure 2A, orange). Under 100 mM KCl conditions, imino proton signals were observed at 10.5–12.0 ppm in the ^1^H-NMR spectrum (Figure 2B, top), indicating the formation of the G4 structure [35]. No signal was observed with 100 mM LiCl (Figure 2B, bottom), indicating no G4 structure. Under LiCl conditions (Figure 2C, open), hORC1^413–511^ showed weaker binding affinity toward teloDNA, with *K*_d_ being 1542.6 ± 76.5 nM, than under KCl conditions (Figure 2C, filled). This clearly indicated that the G4 structure of DNA is critical for strong binding of hORC1^413–511^.

### 2.2. G4 Structures Are Retained in G-Rich DNAs in Complexes with hORC1^413–511^

**Figure 3:**
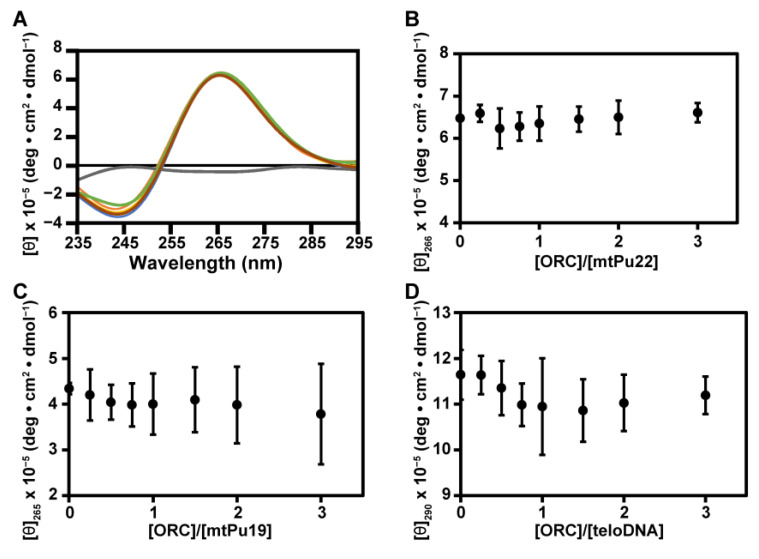
Effects of hORC1^413–511^ upon titration on structures of G-rich DNAs monitored by CD. (**A**) CD spectra of the complex at molar ratios of [hORC1^413–511^]/[mtPu22] = 0.0 (blue), 0.5 (brown), 1.0 (yellow), 1.5 (green), and 2.0 (orange). CD spectrum of hORC1^413–511^ is in gray. (**B**–**D**) The values of (θ) are plotted against [hORC1^413–511^]/[G-rich DNA] for mtPu22 (**B**), mtPu19 (**C**), and teloDNA (**D**). All experiments were performed in duplicate and the standard deviation is indicated for each point.

To gain further insight into the interaction of G-rich DNAs with hORC1^413–511^, we recorded the CD spectra of G-rich DNAs in the presence of various concentrations of hORC1^413–511^ (Figure 3A for mtPu22 as a representative). A positive peak at 266 nm was observed for mtPu22. The appearance of this peak indicates the formation of a parallel-type G4 structure [31]. The intensity of the positive peak at 266 nm was plotted against the molar ratio of [hORC1^413–511^]/[mtPu22] (Figure 3B). The peak intensity did not change with increasing molar ratio. This indicated that mtPu22 retains the G4 structure even in the complex with hORC1^413–511^ and that the binding of hORC1^413–511^ does not unfold the G4 structure.

For mtPu19, the intensity of the positive peak at 265 nm, the appearance of which indicates the formation of a parallel-type G4 structure [31], did not change with increasing molar ratio of [hORC1^413–511^]/[mtPu19] (Figure 3C). This indicated that mtPu19 also retained the G4 structure even in the complex with hORC1^413–511^.

For teloDNA, the intensity of the positive peak at 290 nm, the appearance of which indicates the formation of a (3+1)-type G4 structure as described above, did not change with increasing molar ratio of [hORC1^413–511^]/[teloDNA] (Figure 3D). This indicated that teloDNA also retained the G4 structure, even in the complex with hORC1^413–511^. These results revealed that the G4 structures of all three G-rich DNAs examined are retained even in the complexes with hORC1^413–511^.

**Figure 4:**
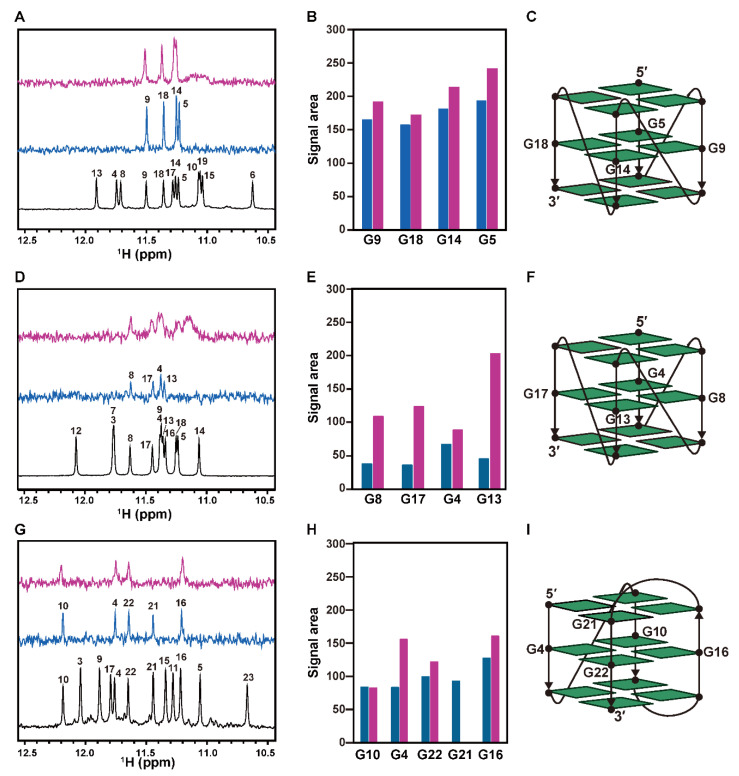
NMR-detected hydrogen-deuterium (H/D) exchange of imino protons of G-rich DNAs in a free form and a complex form with hORC1^413–511^. Imino proton signals of mtPu22 (**A**), mtPu19 (**D**), and teloDNA (**G**) in a free form in H_2_O (black) and ^2^H_2_O (blue) solutions, and in a complex form with hORC1^413–511^ in a ^2^H_2_O solution (red) at pH 7.7 and 25 °C. The assignments were derived from references [30,31,32]. The spectra in the ^2^H_2_O solution were recorded 1.5 h after dissolution of the lyophilized sample in the ^2^H_2_O solution. The signal areas were calculated for mtPu22 (**B**), mtPu19 (**E**), and teloDNA (**H**) in the free (blue) and complex (red) forms. A schematic illustration of each structure is shown for mtPu22 (**C**), mtPu19 (**F**), and teloDNA (**I**).

The retention of the G4 structure in each complex was confirmed by NMR-detected hydrogen-deuterium (H/D) exchange experiments. mtPu22 gave twelve imino proton signals under 100 mM KCl conditions in a H_2_O solution (Figure 4A, bottom). Each imino proton signal originates from one of the twelve guanines involved in the G-tetrad of the G4 structure. Assignments were cited from reference [30]. A sample was lyophilized and dissolved in ^2^H_2_O. Four imino proton signals originating from guanine residues located in the internal G-tetrad were observed (Figure 4A, middle). A schematic illustration of the G4 structure of mtPu22 is shown in Figure 4C. The result indicated that H/D exchange is prohibited for imino protons located in the internal G-tetrad. We supposed that this is due to bulk water not accessing the imino protons located in the internal G-tetrad. mtPu22 in a complex with an equimolar amount of hORC1^413–511^ was prepared under 100 mM KCl conditions in a H_2_O solution, lyophilized, and then dissolved in ^2^H_2_O. An imino proton spectrum of this complex was obtained (Figure 4A, top). Four imino proton signals originating from guanine residues located in the internal G-tetrad were similarly observed. The intensity was also similar for mtPu22 in free and complex forms (Figure 4B). If the G4 structure is unfolded upon binding of hORC1^413–511^, the G-tetrad structure of the G4 structure is destroyed and all imino protons of G residues are exposed to the bulk, resulting in the disappearance of all imino proton signals. Observation of the same four imino proton signals for the complex indicated that the G4 structure is not unfolded on binding of hORC1^413–511^, but that the same G4 structure is retained in the complex.

The imino proton signals originating from guanine residues located in the internal G-tetrad were commonly observed in the ^2^H_2_O solution for free and complex forms for mtPu19 and teloDNA (Figure 4D,E,G,H). These results indicated that the G4 structure is also retained in the complex for mtPu19 and teloDNA.

For mtPu19, the intensities of imino proton signals in a ^2^H_2_O solution in the complex form seemed larger than those in the free form (Figure 4E). This may suggest that bound hORC1^413–511^ masks imino protons and prohibits H/D exchange to some extent in a ^2^H_2_O solution.

### 2.3. Interaction of hORC1^413–511^ with External G-Tetrad Planes of the G4 Structure

**Figure 5:**
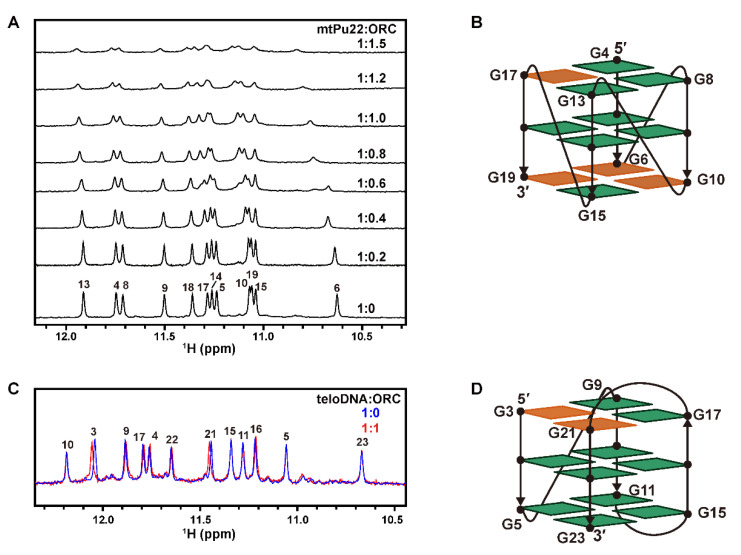
Effects of hORC1^413–511^-binding on ^1^H NMR spectra of mtPu22 and teloDNA. (**A**) Imino proton signals of mtPu22 at each molar ratio of [mtPu22]: [hORC1^413–511^] are shown. (**B**) Schematic illustration of the parallel G4 structure of mtPu22 derived from reference [30]. The residues that exhibited larger chemical shift perturbation (CSP) upon binding with hORC1^413–511^ are highlighted in orange. (**C**) Imino proton signals of teloDNA in free (blue) and complex forms with an equimolar amount of hORC1^413–511^ (red). (**D**) Schematic illustration of the (3+1)-type G4 structure of teloDNA derived from reference [32]. The residues that exhibited larger CSP upon binding with hORC1^413–511^ are highlighted in orange.

To identify the interaction sites of DNAs having G4 structures with hORC1^413–511^, NMR spectra of G-rich DNAs in the presence of various concentrations of hORC1^413–511^ were recorded and chemical shift perturbation (CSP) was analyzed. In the absence of hORC1^413–511^, mtPu22 gave twelve imino proton signals originating from three G-tetrads (Figure 5A, bottom). mtPu22 in a complex with an equimolar amount of hORC1^413–511^ also produced twelve imino proton signals (Figure 5A). This further confirmed that the G4 structure is retained even in the complex.

With increasing molar ratios of [hORC1^413–511^]/[mtPu22], the G6, G10, G17, and G19 signals exhibited significant CSP (Figure 5A). These residues are supposed to be the primary interaction sites for hORC1^413–511^. Figure 5B shows a schematic illustration of the G4 structure of mtPu22 [30]. The residues that exhibited large CSP are colored orange. This suggested that guanine residues located in the external G-tetrads primarily interact with hORC1^413–511^.

For teloDNA, CSP was mainly observed for G3 and G21 (Figure 5C). Figure 5D shows a schematic illustration of the G4 structure of teloDNA, with G3 and G21 colored orange [32]. This suggested again that guanine residues located in the external G-tetrads primarily interact with hORC1^413–511^.

## 3. Discussion

In our previous study, we demonstrated that hORC1^413–511^ binds to artificial G-rich DNAs that form a G4 structure [27]. Here we demonstrated for the first time that hORC1^413–511^ binds to naturally occurring G-rich DNAs, derived from *c-myc* promoter and telomere regions, which form G4 structures, either parallel- or (3+1)-type G4 structures (Figure 1). The affinity toward DNA with a G4 structure was shown to be higher than that toward dsDNA (Figure 1). The importance of the G4 structure for binding was revealed by the affinity decreasing upon destruction of the G4 structure (Figure 2). This implies that the G4 structure of a DNA may be used for the recognition of G-rich DNAs present in the *c-myc* promoter region by hORC for the initiation of replication. The results of our physicochemical analysis are consistent with a growing number of molecular biological and biochemical results suggesting the importance of the G4 structure for replication initiation, as described in the Introduction [4,24,25,26]. Notably, the formation and destruction of the G4 structures of the G-rich DNAs of interest were directly and clearly demonstrated by NMR and CD in this study, which allows us to draw a decisive conclusion as to the importance of the G4 structures for the recognition of these DNAs by hORC1^413–511^.

We previously reported that the G4 structure of a telomere DNA was retained on binding of fused in sarcoma (FUS) [36], while it was destroyed on binding of heterogeneous nuclear ribonucleoprotein D0 (hnRNPD0) [37,38]. Therefore, there are two different scenarios, either retention or destruction of the G4 structure on binding of the protein. Here, we showed that the G4 structure is retained even on binding of hORC1^413–511^ (Figure 3, Figure 4 and Figure 5). Again, NMR and CD provided direct and clear information on this issue. The deduced retention of the G4 structure implies that the G4 structure of a DNA may be used even after the initial recognition by hORC.

Recently, it was reported that ORC of *Drosophila melanogaster* (*Dm*ORC) underwent liquid-liquid phase separation (LLPS) upon binding to a 60 base pair dsDNA [39]. This study revealed that the N-terminal region of *Dm*ORC1, which contains a portion corresponding to hORC1^413–511^ is essential for LLPS. In our present study, LLPS of hORC1^413–511^ was not observed upon binding to G-rich DNAs. Further study is needed to elucidate the possible link of the function of hORC with LLPS.

We identified the interaction sites of DNAs having the G4 structure with hORC1^413–511^. We revealed that the guanine residues located in the external G-tetrads are primary interaction sites. This is the first report of residue resolution for the interaction between the G4 structure of a DNA and hORC1^413–511^. hORC1^413–511^ can interact with the G4 structure independent of the folding, either with a parallel- or (3+1)-type G4 structure, as described above. The identified mode through which hORC1^413–511^ interacts with the guanine residues located in the external G-tetrads may rationalize the fold-independent interaction. The interaction with guanine residues located in external G-tetrads suggests a stacking interaction between the guanine bases and an aromatic ring of F511, which is an only aromatic residue of hORC1^413–511^ and/or the guanidium groups of the arginine residues of hORC1^413–511^.

Our studies suggested that the G4 structure may play a crucial role in the recognition of replication origins by hORC and the subsequent initiation of replication.

## 4. Materials and Methods

### 4.1. Protein Expression and Purification

Previously, the gene encoding hORC1^413–511^ was subcloned into plasmid pGEX-6P-1 (GE Healthcare, Chicago, Illinois, United States) to obtain pGEX-6P-1_hORC1^413–511^, which produces N-terminally glutathione S-transferase (GST)-fused protein GST-hORC1^413–511^ [27]. We transformed *Escherichia coli* BL21-gold (DE3) (Agilent Technologies, Santa Clara, California, United States) with this plasmid. The *E. coli* cells were inoculated into 1L LB medium containing 50 µg/mL ampicillin and grown at 37 °C until the OD value at 600 nm (OD_600_) reached 0.6. Protein expression was induced by adding isopropyl β-1-thiogalactopyranoside (IPTG) to a final concentration of 1 mM and the culture was further incubated at 16 °C. After 24 hours, the culture was cooled on ice and then the cells were harvested by centrifugation at 3000 ×*g* for 20 min at 4 °C. The cell pellet was resuspended in and washed with ice-cold phosphate-buffered saline (PBS), and then centrifuged and resuspended in 40 mL lysis buffer (1× PBS, 1% Triton X-100, 1 mM DTT, 250 mM NaCl, 3 mM MgCl_2_, 10 mM benzamidine, and 1.2 mg/mL lysozyme). This suspension was stirred gently at room temperature for 20 min, and then treated with (12.5 U/mL) DNase I and (10 µg/mL) RNase A for another 20 min. The supernatant was collected by ultracentrifugation at 50,000× *g* for 20 min at 4 °C and filtered using a 0.45 μm filter (Membrane Solutions Limited, Plano, Texas, United States). This solution was applied to a glutathione-Sepharose column, which was equilibrated with AB (1× PBS, 250 mM NaCl, and 10 mM benzamidine). The protein-bound column was washed extensively with AB. Subsequently, AB supplemented with 10 µg/mL RNase A was applied to the column. After incubation for 2 hours, the column was washed with AB extensively. Then, BB (20 mM HEPES-KOH (pH 7.7), 100 mM KCl, 1 mM EDTA-2Na, and 1 mM DTT) supplemented with PreScission protease (N-terminally GST-tagged) was applied to the column, followed by incubation for 12 hours, and GST-cleaved hORC1^413–511^ was eluted. The purified protein was stored at 4 °C. The protein concentration was determined from the UV absorbance at 214 nm using a molar extinction coefficient (ɛ) value (147,978 M^-1^ cm^-1^) by a method described in reference [40]. A bicinchoninic acid (BCA) assay was also carried out using a kit, Pierce™ BCA Protein Assay Kit—Reducing Agent Compatible (Thermo Fisher Scientific, Waltham, Massachusetts, United State).

### 4.2. Preparation of DNAs

The DNAs used in the present study are listed in Table 1. All the DNAs and fluorescein (FAM)-labeled DNAs were synthesized, purified, and de-salted by FASMAC Co., Ltd. (Kanagawa, Japan).

### 4.3. Fluorescence Anisotropy (FA) Measurement

The FAM-labeled DNAs were dissolved in BB or BB containing 100 mM LiCl instead of 100 mM KCl (BB_LiCl_). They were heated at 95 °C for 5 min, after which wtPu22, wtPu19, mtPu22, and mtPu19 were cooled rapidly on ice, and teloDNA and dsDNA were cooled gradually at a rate of 1 °C/min. For the titration experiments under LiCl conditions, hORC1^413–511^ in BB was dialyzed against BB_LiCl_. hORC1^413–511^ was titrated against DNA (2 μM) to obtain a range of molar ratios of [hORC1^413–511^]/[FAM-labeled DNA] = 0.0–2.0. FA was recorded at 25 °C using a FP-8500 spectrofluorometer (JASCO, Hachioji, Tokyo, Japan). FAM-labeled DNAs were excited at 485 nm and FA was recorded at 520 nm. The dissociation constant of a complex (*K*_d_) was deduced by curve fitting using the following equations, assuming a 1:1 binding mode [41]

(1) $$m=1+\left(\frac{P}{D_{0}}\right)+\left(\frac{K_{d}}{D_{0}}\right)$$

(2) $$A=A_{f}-\left(A_{f}-A_{b}\right)\left(0.5m-\sqrt{0.25m^{2}-\frac{P}{D_{0}}}\right)$$

where *P* is the total concentration of hORC1^413–511^, *A* is the measured anisotropy, *A*_f_ is the anisotropy value of the free DNA, *A*_b_ is the maximum anisotropy value obtained when the DNA is fully bound to hORC1^413–511^, and *D*_0_ is the total DNA concentration.

### 4.4. CD Spectroscopy

The mtPu22, mtPu19, and teloDNAs were dissolved in BB or BB_LiCl_. They were heated and cooled as described above. CD measurements were carried out using individual 5 μM DNA solutions with 0–3 molar ratios of hORC1^413–511^. CD spectra were recorded using a J-720 spectropolarimeter (JASCO, Hachioji, Tokyo, Japan).

### 4.5. NMR Spectroscopy

The DNAs were dissolved in BB or BB_LiCl_ supplemented with 10% D_2_O and 10 µM 4,4-dimethyl-4-silapentane-1-sulfonic acid (DSS). The DNAs were heated and cooled as described above. NMR titration of the hORC1^413–511^ against 75 μM DNAs was performed at 25 °C. The hORC1^413–511^ solution was added to the DNA solution to obtain molar ratios [hORC1^413–511^/DNA] = 0, 0.2, 0.4, 0.6, 0.8, 1.0, 1.2, and 1.5. All NMR spectra were recorded on a Bruker Avance III HD 600 spectrometer equipped with a cryogenic probe and Z-gradient.

### 4.6. NMR-Detected Hydrogen–Deuterium Exchange (H/D Exchange) Measurement

The DNAs were dissolved in BB supplemented with 10 µM DSS, and then heated and cooled as described above. The protonated solutions of 50 μM DNAs and 50 μM DNAs mixed with equimolar hORC1^413–511^ were lyophilized and dissolved in ^2^H_2_O 1.5 hours before the NMR experiments. 1D ^1^H NMR spectra were recorded at 25 °C using a Bruker Avance III HD 600 spectrometer equipped with a cryogenic probe and Z-gradient. The same number of scans and receiver gain were applied for all samples.

## Figures and Tables

**Table 1:** Sequences of DNAs used in this study.

Name	Sequence	Structure
mtPu22	5′-TGAGGGTGGGTAGGGTGGGTAA-3′	Parallel G4
mtPu19	5′-TAGGGAGGGTAGGGAGGGT-3′	Parallel G4
teloDNA	5′-TTGGGTTAGGGTTAGGGTTAGGGA-3′	(3+1)-type G4
wtPu22	5′-TGAGGGTGGGGAGGGTGGGGAA-3′	G4
wtPu19	5′-TGGGGAGGGTGGGGAGGGT-3′	G4
dsDNA_1	5′-AAATTTAAAAAAAAAAATAATT-3′	Duplex
dsDNA_2	5′-AATTATTTTTTTTTTTAAATTT-3′	

Fluorescein (FAM) is attached to the 5′-end of each DNA except for dsDNA_2.

## Data Availability

Not applicable.
